# *Mycobacterium bovis* Bacille Calmette-Guerin infection modulates GRK2/3 dependent cytokine secretion

**DOI:** 10.1186/1471-2334-14-S3-E20

**Published:** 2014-05-27

**Authors:** Manisha Yadav, D Nagarjuna, Rakesh Singh Dhanda

**Affiliations:** 1 Dr. B.R. Ambedkar Centre for Biomedical Research, University of Delhi, Delhi, India; 2 Department of Translational and Regenerative Medicine, Postgraduate Institute of Medical Education and Research, Chandigarh, India

## Background

*Mycobacterium tuberculosis* has evolved highly specialized mechanisms to proliferate in the host during infection. In this process, the infection of alveolar epithelial cells is a necessary step for mycobacteria dissemination; however the mechanisms of mycobacterial epithelial interactions are incompletely understood. Previously, we characterized the role of epithelial G protein coupled receptors (GPCR) CXCR1 and CXCR2 during mycobacterial infection. However the role of GPCR kinases (GRK) 2/3 and GRK4-6 in response to mycobacterial infection has not been investigated.

## Methods

The GPCR kinases expression (GRK2/3 and GRK4-6) after Mycobacterium infection was quantified by RT-PCR and Western blot analysis. Further, the secretion of cytokines IL-8 and TNF-α was quantified in supernatants by ELISA.

## Results

Mycobacterial infection in lung epithelial cells increased secretion of IL-8 and decreased TNF-α upto 72 hours. Further, the infection in the epithelial cells was modulated by a combined up regulation of GPCR kinases (*GRK*) 2/3 genes and suppression of the *GRK* 4-6 gene expression. These results were confirmed at protein levels. In addition, the blocking of chemokine receptors decreased the inhibition of *GRK* 2/3 expression suggesting that mycobacteria manipulate epithelial responses by desensitizing the receptors and the cytokine secretion.

## Conclusions

In conclusion, we have identified a role for *GRK* 2/3 dependent cytokine secretion in the initial phase of mycobacterial infections in the lung epithelial cells.

